# Istradefylline prevents central nervous system oxygen toxicity

**DOI:** 10.3389/fnmol.2026.1922859

**Published:** 2026-10-23

**Authors:** Zongkai Du, Ruhan Li, Yue Jiang, Yanan Zhang, Chenggang Zheng, Runping Li

**Affiliations:** 1 PLA Naval Medical Center, Naval Medical University, Shanghai, China; 2 Department of Military and Special Medicine, No. 971th Hospital of PLA Navy, Qingdao, China; 3 Qingdao Medical College, Qingdao University, Qingdao, China; 4 Department of Hyperbaric Oxygen Therapy, Shanghai Changhai Hospital, Shanghai, China

**Keywords:** adenosine A2A receptor antagonist, central nervous system oxygen toxicity, istradefylline, oxygen convulsions, prophylactic effect

## Abstract

Central nervous system oxygen toxicity (CNS-OT) is a toxic reaction induced by inhalation of gases with excessively high partial pressure of oxygen, for which effective proactive preventive and therapeutic measures are currently lacking. Building on our previous work implicating adenosine A2A receptor (A2AR) signaling, we examined the prophylactic effects of istradefylline (KW-6002), an approved A2AR antagonist, in male mice. We found that pretreatment oral administration of istradefylline (0.5–20 mg/kg) in mice significantly prolonged the latency to seizure onset and markedly reduced both the intensity and frequency of seizures during a 30-min exposure to 100% oxygen at 5 ATA. Cortical targeted metabolomics showed that HBO exposure decreased GABA and increased glutamate; istradefylline increased cortical GABA without significantly reducing cortical glutamate. Separate ELISA measurements showed lower hippocampal glutamate in istradefylline-treated mice than in HBO-exposed vehicle controls, whereas reductions in the other regions examined were not statistically significant. In the striatum, istradefylline was associated with lower cAMP, immunoreactive PKA protein concentration, and phosphorylated CREB, together with reduced GluA1 Ser845 phosphorylation and increased GABA_A receptor β3 Ser408/409 phosphorylation. In the cortex, malondialdehyde levels decreased and catalase activity increased, whereas glutathione peroxidase activity decreased further. Striatal IL-1β, IL-6, and TNF-α mRNA levels were also reduced. These findings support a prophylactic effect of istradefylline against HBO-induced seizures in male mice and identify associated changes in neurotransmitter levels, receptor phosphorylation, signaling markers, oxidative-stress measures, and inflammatory-gene expression. These results provide a theoretical basis for the development of proactive preventive and therapeutic strategies for CNS-OT with potential clinical application.

## 1 Introduction

Central nervous system oxygen toxicity (CNS-OT) refers to a series of toxic reactions in the central nervous system that occur after the body inhales oxygen above a certain pressure for an extended duration. It primarily occurs during military and civil diving, hyperbaric operations, and hyperbaric oxygen therapy. When the partial pressure of inhaled oxygen exceeds 280 kPa (equivalent to a water depth of 18 meters), toxic reactions occur within a short period, and as the pressure increases, the latency to onset further shortens. During onset, the most severe reaction manifests as generalized tonic–clonic seizures, known as “oxygen-induced seizures” or “oxygen convulsion” (Ciarlone et al., 2019), which poses a serious threat to the life and health of individuals exposed to hyperbaric oxygen, severely constraining the efficiency of diving and hyperbaric operations, as well as the potential efficacy of therapeutic applications. Currently, there is a lack of effective, proactive preventive measures for CNS-OT. Management primarily relies on passive strategies, such as limiting the pressure and duration of oxygen exposure, including the use of intermittent oxygen breathing. Identifying active measures to prevent oxygen convulsions, thereby enabling the safe use of oxygen at higher pressures (greater depths), is of paramount importance for diving, hyperbaric operations, and hyperbaric oxygen therapy (HBOT).

In recent years, the role of the adenosinergic system in HBOT and oxygen toxicity has garnered widespread attention (You et al., 2025; Boussuges et al., 2022; Đambić et al., 2025). Adenosine, a purine nucleoside, is primarily derived from the dephosphorylation of adenosine triphosphate (ATP). Its extracellular concentration is regulated by tissue energy status, oxygenation level, and inflammatory state (Satyam et al., 2026). Adenosine modulates neural functions mainly through the A1, A2A, A2B, and A3 receptors of the G protein-coupled receptor (GPCR) family (Haas and Selbach, 2000; Salmaso et al., 2025). Under physiological conditions, adenosine primarily binds to the high-affinity A1 receptors (A1R), inhibiting presynaptic voltage-gated calcium channels and reducing the release of the excitatory neurotransmitter glutamate, thereby diminishing neuronal excitability and exerting a central inhibitory effect (Salmaso et al., 2025; McCool and Farroni, 2001). Conversely, under pathological conditions, the expression of A2A receptors (A2AR) in astrocytes and microglia is significantly upregulated, which promotes neuroinflammatory responses and further exacerbates the risk of neurodegeneration and seizures (Gao, 2023). Currently, the development of drugs targeting A2AR for the treatment of neurodegenerative diseases, such as Parkinson’s disease (PD), has gained increasing attention (Pinna et al., 2018; Ferré et al., 2018). In the basal ganglia, A2AR are primarily expressed on gamma-aminobutyric acid (GABA)ergic medium spiny neurons (MSNs) in the striatum. There A2AR form heterodimers with dopamine D2 receptors (D2R) on MSNs, which are coupled to functionally opposing excitatory G proteins (Gs) and inhibitory G proteins (Gi), respectively. This coupling establishes functionally antagonistic receptor dimers that synergistically regulate neurotransmission and motor control (Ferré et al., 2018; Fuxe et al., 2010). In PD, overactivation of A2AR exacerbates symptoms associated with dopamine deficiency. Antagonizing A2AR improves motor symptoms, thereby effectively reducing rigidity and tremors.
Research has shown that hyperbaric oxygen exposure increases extracellular adenosine levels by inhibiting adenosine kinase (ADK) activity and enhancing 5′-nucleotidase activity (Chen et al., 2016). Our previous study revealed that the A2AR agonist CGS21680, even at ultra-low doses ranging from 0.003–0.1 μg, significantly shortened the latency to oxygen-induced seizures. Furthermore, the activation or inhibition of dopamine D2 receptors did not alter the pro-convulsant effect of CGS21680. Conversely, the A2AR antagonist SCH58261 effectively suppressed oxygen-induced seizures; doses of SCH58261 between 1 and 30 μg significantly prolonged the seizure latency. Similarly, the concomitant activation or inhibition of dopamine D2 receptors exerted no significant impact on this protective prolongation (You et al., 2025). The precise role of A2AR in the pathogenesis of CNS-OT remains to be fully elucidated.

Given the multifaceted roles of the A2AR in neuroexcitatory transmission, oxidative stress, and motor regulation, we hypothesize that A2AR may function as a critical “molecular switch” in CNS-OT. Consequently, its functional antagonists hold significant potential for development as therapeutic agents for the prevention of CNS-OT. Istradefylline (KW-6002), a potent and highly selective A2AR antagonist developed by Kyowa Kirin Co., Ltd., received approval from the Pharmaceuticals and Medical Devices Agency (PMDA) of Japan in March 2013 under the trade name Nouriast. Subsequently, in August 2019, the U. S. Food and Drug Administration (FDA) also approved its clinical use as an add-on treatment to medopa in patients with Parkinson’s disease experiencing “off” episodes (Paton, 2020; Chen and Cunha, 2020). Our previous studies examined whether pharmacological modulation of adenosine A2A receptors could protect against CNS-OT, but did not systematically characterize the molecular changes associated with this protection. In the present study, we evaluated the prophylactic effect of istradefylline, an A2A receptor antagonist approved as adjunctive therapy for Parkinson’s disease, in mice exposed to hyperbaric oxygen (HBO). We measured neurotransmitter levels, cAMP/CREB-related markers, oxidative-stress indices, and pro-inflammatory cytokine mRNA expression in selected brain regions. We hypothesized that istradefylline pretreatment would attenuate HBO-induced behavioral seizures and be accompanied by changes in these measured molecular endpoints.

## 2 Materials and methods

### 2.1 Materials

#### 2.1.1 Experimental animals

Male C57BL/6 mice (weight: 22–26 g) were obtained from Shanghai Sippr-BK Laboratory Animal Co., Ltd. (License No. SCXK (Shanghai) 2022–0001). All animals were acclimated to a clean-grade housing environment for one week prior to the commencement of experiments. To minimize the influence of circadian rhythms, all experimental procedures were strictly conducted between 09:00 and 12:00. The housing environment was maintained at a temperature of 22 ± 2 °C and a humidity of 50 ± 5%, with a 12-h light/dark cycle, and ad libitum access to food and water. All animal procedures were reviewed and approved by the Institutional Animal Care and Use Committee (IACUC) of the Naval Medical University (Approval No. IACUC-2023-045) and were in strict accordance with the “Regulations on the Management of Laboratory Animals.”

#### 2.1.2 Reagents and equipment

Istradefylline (KW-6002, HY-10888) was purchased from MedChemExpress (MCE, USA). Enzyme-linked immunosorbent assay (ELISA) kits for gamma-aminobutyric acid (GABA, MM-9268430), glutamate (Glu, MM927086), phosphorylated cAMP-response element-binding protein (p-CREB, MM-0785 M1), protein kinase A (total PKA, MM-44733 M1), and cyclic adenosine monophosphate (cAMP, MM-0544 M1) were obtained from Jiangsu Meimian Industrial Co., Ltd. (Jiangsu, China). The following primary antibodies were purchased from Abcam (Cambridge, MA, USA): recombinant anti-glutamate receptor 1 (AMPA subtype, phospho-S845, 1:200, ab222761), recombinant anti-glutamate receptor 1 (AMPA subtype, 1:1000, ab183797), anti-GABAA receptor β3/GABRB3 (phospho-S408 + S409, 1:1000, ab254130), and anti-GABAA receptor β3/GABRB3 (1:1000, ab98968). Biochemical assay kits for glutathione peroxidase (GPx), catalase (CAT), and malondialdehyde (MDA) were purchased from Beyotime Institute of Biotechnology (Shanghai, China).

### 2.2 Methods

#### 2.2.1 Experimental grouping

In the study investigating the effects of KW-6002 on oxygen-induced seizures, mice were randomly assigned to seven groups: Control group (*n* = 7), 0.5 mg/kg KW-6002 group (*n* = 8), 1 mg/kg KW-6002 group (*n* = 8), 2.5 mg/kg KW-6002 group (*n* = 7), 5 mg/kg KW-6002 group (*n* = 7), 10 mg/kg KW-6002 group (*n* = 6), and 20 mg/kg KW-6002 group (*n* = 6).

To explore the underlying mechanisms by which KW-6002 influences oxygen-induced seizures, mice were randomly divided into three groups: a Normal group (no HBO exposure), a Control group (HBO exposure following vehicle DMSO administration), and a KW-6002 group (HBO exposure following KW-6002 administration). Drugs were administered via oral gavage (10 mL/kg), and the dose of KW-6002 was 5 mg/kg.

Mice were allocated to prespecified group sizes by sorting Excel-generated random numbers. Seizure scoring, subsequent assays, and data analysis were performed by investigators blinded to treatment allocation.

#### 2.2.2 Preparation of KW-6002

KW-6002 (10 mg) was accurately weighed and dissolved in 1 mL of DMSO with vortex mixing until complete dissolution, achieving a concentration of 10 mg/mL; the solution was stored protected from light at 4 °C. The stock solution was then diluted with 9 mL of corn oil to a final volume of 10 mL, yielding a working solution of 1 mg/mL. The vehicle (1:9, DMSO: corn oil, v/v) was prepared by mixing 1 mL of DMSO with 9 mL of corn oil and vortexing thoroughly.

#### 2.2.3 CNS-OT induction and seizure scoring

One hour after oral gavage, mice were placed in a small-animal hyperbaric chamber (RDC150-300-6, Naval Medical University, Shanghai, China). Soda lime was placed at the bottom of the chamber to absorb CO_2_, and the ambient temperature was maintained at 22–26 °C. The chamber was flushed with pure oxygen at a flow rate of 1 L/min for 3 min to replace the internal air, followed by pressurization to 5 Atmosphere Absolute (ATA) at a rate of 1 ATA/min (Figure 1A). Behavioral changes were closely monitored; generalized tonic–clonic seizures (GTCS), characterized by classic manifestations such as exhibited two or more of the following symptoms: head bobbing up and down, blinking, elevation of one or two front paws, most often with opened toes or pushing the ground, or multiple and intense shakes or thrashing, were identified as the endpoint according to established behavioral criteria for CNS-OT (Domachevsky et al., 2013). The seizure latency was recorded as the time interval from reaching 5 ATA to the onset of the first oxygen-induced seizures. The total duration of HBO exposure was 30 min, during which the frequency of seizures was recorded, and seizure severity was graded on a 0–3 scale (Ari et al., 2019): Grade 0: no tonic–clonic seizures; Grade 1: mild and brief seizures (< 10 s) without postictal symptoms; Grade 2: intense but brief seizures (< 10 s) or mild but prolonged seizures (> 10 s); Grade 3: severe and prolonged seizures (> 10 s) with extensive postictal exhaustion. Two observers blinded to treatment independently scored the recordings; disagreements were resolved by a third blinded observer before treatment codes were disclosed. Following HBO exposure, the chamber was decompressed to atmospheric pressure at a rate of 1 ATA/min. All mice underwent the planned 30-min HBO exposure regardless of seizure occurrence. Decompression then took approximately 4 min, after which the mice were promptly anesthetized and euthanized; brain tissue was collected within 10 min of euthanasia. Mice were anesthetized with isoflurane and transcardially perfused with phosphate-buffered saline (PBS). The cortex, hippocampus, and striatum were rapidly dissected on ice, snap-frozen in liquid nitrogen, and stored at −80 °C for subsequent analysis.

**Figure 1:**
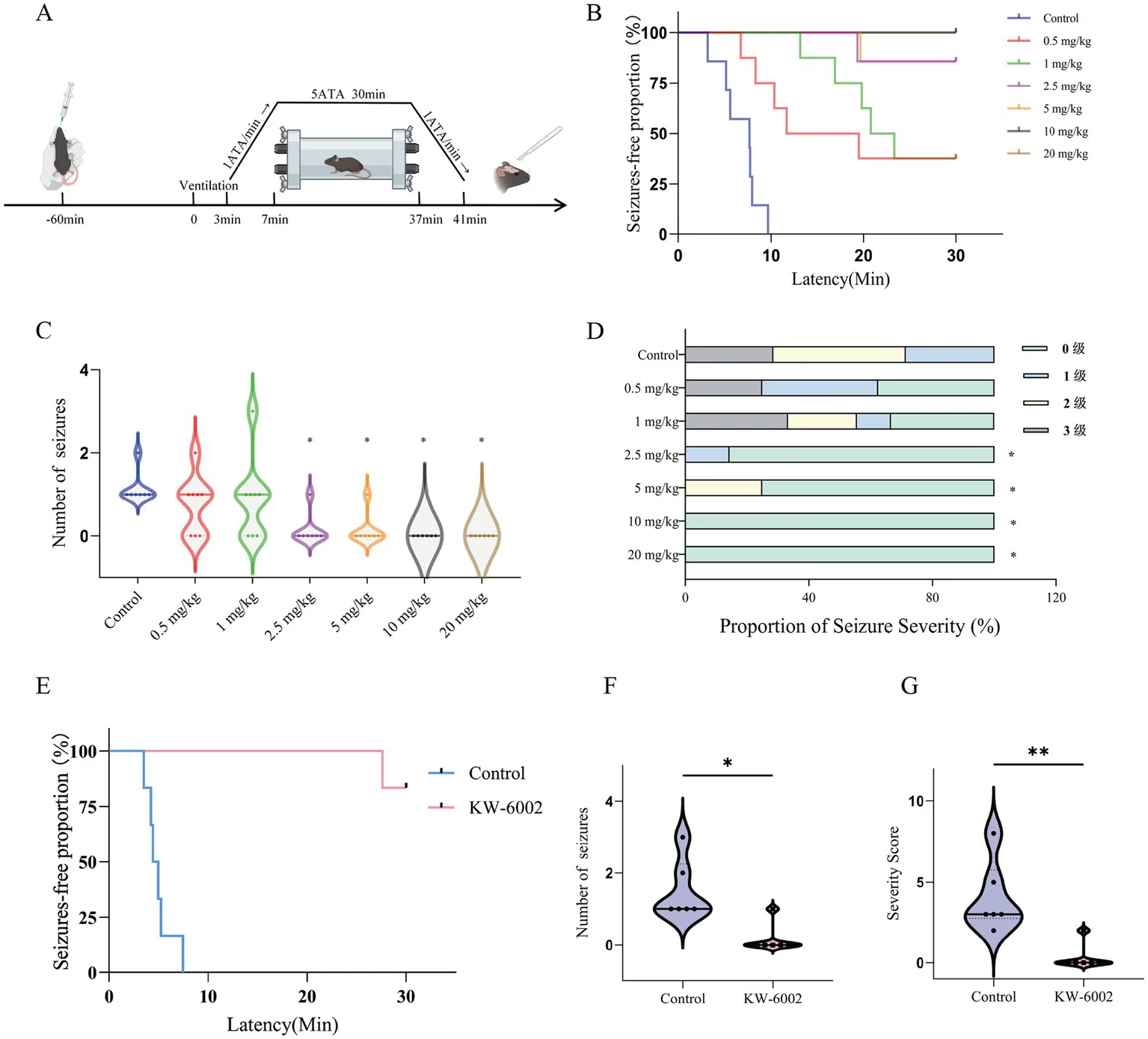
Effects of KW-6002 on oxygen-induced seizures. **(A)** Ex*p*erimental flowchart. In the Control group and 0.5–20 mg/kg KW-6002 groups, **(B)** Seizure-free proportion; **(C)** Number of seizures; **(D)** Proportion of seizure severity. In the Control and 5 mg/kg KW-6002 groups, **(E)** Seizure-free proportion **(F)** Number of seizures; **(G)** Severity scores. ^*^p < 0.05; ^**^p < 0.01 vs. Control group (*n* = 6). All data are presented as mean ± SD.

Each HBO exposure run included two to three mice from each HBO-exposed group in the same chamber. Mice moved freely without physical partitions, were individually identified, and had their outcomes recorded separately. Chamber operation, gas delivery, and animal handling followed the same standardized protocol across runs. Including both HBO-exposed groups in each run reduced confounding between treatment and run conditions. For comparisons of pharmacological treatments, the individual mouse was the unit of treatment allocation and analysis.

#### 2.2.4 Targeted Metabolomic analysis of neurotransmitters

Cortical tissues were collected from the Normal, Control, and KW-6002 groups (*n* = 6 per group) for targeted metabolomic quantification of neurotransmitters. The analysis was conducted by Novogene Co., Ltd. (Beijing, China). Absolute quantification of specific metabolites was performed using a SCIEX QTRAP 5600 + mass spectrometer, with authentic standards used as references. The analytical workflow integrated sample collection, metabolite extraction, standard curve construction, methodological validation, and LC–MS detection. To evaluate linearity, a series of standard solutions were analyzed, and calibration curves were generated by plotting the ratio of the peak area of the standard to the internal standard against their respective concentration ratios. The limit of quantification (LOQ) was determined based on the signal-to-noise (S/N) ratio method, where the LOQ was defined as the concentration corresponding to an S/N ratio of 10:1.

#### 2.2.5 Western blotting

Striatal tissues samples were homogenized in sterile ice-cold PBS (1 mL/100 mg tissue) using a low-temperature tissue grinder (*n* = 6 per group). The homogenates were centrifuged at 12,000 × g for 15 min at 4 °C. The supernatant was discarded, and the pellet was resuspended in RIPA lysis buffer (P0038, Beyotime) supplemented with protease and phosphatase inhibitors. After ultrasonic disruption, the samples were incubated on ice for 30 min for complete lysis, with gentle mixing every 5 min. Following another centrifugation at 12,000 × g for 15 min, the supernatant was collected, and protein concentrations were quantified using a BCA Protein Assay Kit (P0010, Beyotime). Protein concentrations were then normalized, and samples were denatured in 5 × SDS loading buffer at 95 °C for 10 min using a metal bath. Equal amounts of protein were separated by SDS-PAGE and transferred onto PVDF membranes using a rapid transfer system (Standard program). Membranes were blocked with 1% bovine serum albumin (BSA) at room temperature for 2 h and then incubated overnight at 4 °C with primary antibodies: β-actin, p-AMPAR, and p-GABAR. After washing with TBST, the membranes were incubated with HRP-conjugated secondary antibodies (1:5,000) for 1 h at room temperature, followed by detection via enhanced chemiluminescence (ECL). To detect total protein levels, the membranes were treated with stripping buffer to remove the original antibodies and then re-probed for total AMPAR and GABAR following the same blocking and incubation procedures.

#### 2.2.6 Enzyme-linked immunosorbent assay (ELISA)

All ELISA procedures were performed in accordance with the manufacturer’s instructions. Striatal tissues samples were homogenized in sterile ice-cold PBS (1 mL/100 mg tissue) using a low-temperature tissue grinder (*n* = 6 per group). The homogenates were centrifuged at 3,000 × g for 10 min, and the resulting supernatants were used for the assays. The PKA ELISA measured immunoreactive PKA protein concentration rather than kinase activity. For the quantification of cAMP, PKA, and p-CREB, a double-antibody sandwich ELISA was employed. Briefly, samples, standards, and horseradish peroxidase (HRP)-conjugated detection antibodies were sequentially added to microwells pre-coated with the specific primary antibody. After incubation and thorough washing, the substrate 3,3′,5,5′-tetramethylbenzidine (TMB) was added for color development. Under the catalysis of peroxidase, the substrate turned blue and subsequently shifted to yellow upon the addition of an acidic stop solution. The optical density (OD) was measured at a wavelength of 450 nm using a microplate reader to calculate sample concentrations. For the detection of GABA and Glu, a competitive ELISA was utilized. Samples, standards, and HRP-conjugated competitive antigens were added to microwells pre-coated with the respective antibody. Following incubation and washing, color development was performed using TMB, and the reaction was terminated with acid as described above. The OD values were measured at 450 nm to determine the concentrations of the target metabolites.

#### 2.2.7 Assessment of oxidative stress markers

All assays were performed in strict accordance with the manufacturer’s instructions (*n* = 6 per group). MDA content determination: The concentration of MDA in the cortex was quantified using a colorimetric assay based on the reaction between MDA and thiobarbituric acid (TBA), which yields a red chromogenic product. CAT activity assay: CAT activity in tissue samples was determined through a chromogenic reaction. A standard curve was constructed using hydrogen peroxide (H_2_O_2_) standards. The absorbance was measured at 520 nm using a UV–visible spectrophotometer. The enzyme activity was calculated by quantifying the amount of H_2_O_2_ catalyzed into water and oxygen by CAT per unit volume and time. GPx activity assay: The activity of selenium-dependent GPx in tissue samples was evaluated using an ultraviolet colorimetric method. The GPx activity levels were calculated by monitoring the rate of NADPH decrease via the change in absorbance at 340 nm.

#### 2.2.8 Quantitative Real-Time PCR (RT-qPCR)

Total RNA was extracted from the striatal tissues using the VAMNE Magnetic Cell/Tissue Total RNA kit (Prepackaged)-BOX1 according to the manufacturer’s protocol, and RNA concentrations were quantified (*n* = 6 per group). The total RNA was reverse-transcribed into cDNA using HiScript III All-in-one RT SuperMix Perfect for qPCR, which contains HiScript III reverse transcriptase. The thermal program: 50 °C for 15 min, followed by 85 °C for 5 s and a 4 °C hold.

The mRNA expression levels of interleukin-1β (*Il1b*), tumor necrosis factor-α (*Tnf-a*), and interleukin-6 (*Il6*) were quantified via RT-qPCR. All primers were synthesized by Sangon Biotech (Shanghai) Co., Ltd., and the sequences are summarized as follows: *Il1b* Forward: 5′-GCAACTGTTCCTGAACTCAACT-3′, Reverse: 5′-ATCTTTTGGGGTCCGTCAACT-3′; *Tnf-a* Forward: 5′-CCCTCACACTCAGATCATCTTCT-3′, Reverse: 5′-GCTACGACGTGGGCTACAGT-3′; I*l6* Forward: 5′-TAGTCCTTCCTACCCCAATTTCC-3′, Reverse: 5′-TTGGTCCTTAGCCACTCCTTC-3′; *Actb* Forward: 5′-GGCTGTATTCCCCTCCATCG-3′, Reverse: 5′-CCAGTTGGTAACAATGCCAT-3′.

β-actin (*Actb*) served as the internal control, and the relative mRNA expression levels were calculated using the 2^−ΔΔCt^ method. The PCR cycling conditions consisted of an initial denaturation at 95 °C for 5 min, followed by 45 cycles of denaturation at 95 °C for 5 s, annealing at 58 °C for 10 s, and extension at 72 °C for 20 s.

### 2.3 Statistical analysis

Data analysis was performed using GraphPad Prism software (version 10.4.0). Quantitative data are presented as the mean ± standard deviation (Mean ± SD). The normality of data distributions was assessed using the Shapiro–Wilk test. Each continuous outcome was analyzed separately within each brain region. Normally distributed outcomes were analyzed using one-way ANOVA, followed by Dunnett’s test when dose groups were compared with the vehicle-treated HBO control or Tukey’s test when all pairwise group comparisons were examined. A *p*-value < 0.05 was considered statistically significant.

For oxygen-induced seizure latency, mice that did not exhibit seizures during the 30-min hyperbaric oxygen exposure were assigned a latency of 30 min (censored data). Survival analysis was conducted using IBM SPSS Statistics (version 27.0); seizure-free probabilities were estimated using the Kaplan–Meier method, and differences between groups were evaluated using the Log-rank test. For data that did not follow a normal distribution, the Kruskal-Wallis non-parametric test was employed.

## 3 Results

### 3.1 KW-6002 attenuates oxygen-induced seizures in a dose-dependent manner

Mice in the KW-6002 groups exhibited no sedative manifestations, such as a marked reduction in locomotor activity, following administration or during hyperbaric oxygen (HBO) exposure. KW-6002, at doses ranging from 0.5–20 mg/kg, effectively delayed the onset of oxygen-induced seizures (Figure 1B), reduced seizure frequency (Figure 1C), and diminished seizure severity (Figure 1D). Notably, in the 2.5 and 5 mg/kg dose groups, the proportion of seizure-free mice was 6/7 (85.7%, only one out of seven mice exhibited a seizure, with a latency exceeding 20 min). Given the individual variability and the fact that the latency was more than two-fold longer than that of the Control group, these results indicate that KW-6002 significantly delays oxygen-induced seizures within this dose range.

To further investigate the mechanisms underlying the effects of KW-6002 on CNS-OT while minimizing potential off-target effects and systemic toxicity associated with higher concentrations, 5 mg/kg was selected as the optimal dose for subsequent experiments. In the mechanistic study, only one mouse in the 5 mg/kg KW-6002 group exhibited a seizure (at approximately 26 min), whereas all six mice in the Control group experienced seizures within the first 10 min of exposure (Figure 1E). In the KW-6002 group, the single affected mouse showed a Grade 2 seizure. In contrast, in the Control group, two mice experienced two and three seizures, respectively, during the 30-min exposure period, representing a significant difference in seizure frequency (Figure 1F, *p* < 0.05). Furthermore, the seizure severity scores in the Control group were significantly higher than those in the KW-6002 group (Figure 1G).

### 3.2 KW-6002 restores the GABA/Glu imbalance in the cerebral cortex

The results of the targeted neurotransmitter metabolomics analysis are summarized (Table 1). Sorting by *P*-value revealed that GABA and Glu were the most significantly altered metabolites. Compared with the Normal group, the GABA content in the Control group was significantly decreased; however, this reduction was reversed by KW-6002 treatment (Figure 2A). While Glu levels were significantly elevated in both the Control and KW-6002 group compared with the Normal group, no significant difference was observed between the two HBO-exposed groups (Figure 2B). The Glu/GABA ratio serves as a critical indicator of the imbalance between excitatory and inhibitory neurotransmission. Our results demonstrated that the Glu/GABA ratio was significantly increased in the Control group relative to the Normal group, an effect that was effectively reversed by KW-6002 (Figure 2C).

**Table 1:** Changes in cortical neurotransmitter levels and metabolic ratios in each group of mice (Mean ± SD; *n* = 6).

Neurotransmitter	Normal	Control	KW-6002	*F*	*P*
γ-aminobutyric acid (GABA)	285654±19374	178056±29431^##^	219721±26668^##*^	27.130	<0.0001
Glutamic acid (Glu)	315699±53371	408595±34739^##^	408941±47308^##^	8.258	0.0038
Tryptophan (Trp)	15573±1594	13214±1284	18208±3876^*^	5.848	0.0133
L-kynurenine (Kyn)	32.73±8.293	53.02±13.25	96.71±64.11^#^	4.419	0.0310
Epinephrine (E)	16.96±1.492	14.93±3.514	20.07±3.564^*^	4.418	0.0310
Picolinic acid (PA)	2.530±1.935	0.6650±0.4456	1.397±0.8443	3.415	0.0600
Dopamine (DA)	3189±657.8	2442±520.5	2514±459.5	3.344	0.0630
Xanthurenic acid (XA)	981.4±102.3	917.9±182.8	1164±267.6	2.536	0.1125
Glutamine (Gln)	349836±35706	380987±62971	408567±34969	2.405	0.1242
Acetylcholine (Ach)	639.8±182.5	439.7±199.3	526±63.4	2.353	0.1292
Histamine (HisA)	61.00±26.75	55.40±33.53	31.27±10.41	2.305	0.1340
Tyrosine (Tyr)	43210±8029	35104±6140	39010±7293	1.904	0.1833
Kynurenic acid (KynA)	6.317±1.341	5.553±1.985	8.240±3.528	1.897	0.1843
Melatonin (MT)	5.280±2.392	6.150±2.189	3.982±1.445	1.701	0.2159
5-hydroxytryptamine (5-HT)	113.5±40.10	187.0±93.52	124.6±87.10	1.576	0.2393
5-Hydroxytryptophan (5-HTP)	11.83±3.31	5.24±3.81	18.23±26.85	1.017	0.3852
Levodopa (DOPA)	889.1 ±77.50	768.0±212.5	821.8±203.7	0.716	0.5048
Histidine (His)	21626±5452	19653±3986	21033±3465	0.320	0.7308
Norepinephrine (NE)	1391±323.2	1404±454.4	1460±385.9	0.052	0.9493
GLU/GABA	1.113±0.223	2.345±0.396^##^	1.890±0.362^##^	20.54	<0.0001
KynA/Kyn	0.200±0.045	0.1054±0.025^##^	0.102±0.057^##^	9.467	0.0022
Kyn/Trp	0.002±0.001	0.004±0.001	0.005±0.003^#^	5.333	0.0178

Compared with normal group, ^#^*P* < 0.05, ^##^*P* < 0.01; compared with Control group; *P < 0.05; **P < 0.01.

**Figure 2:**
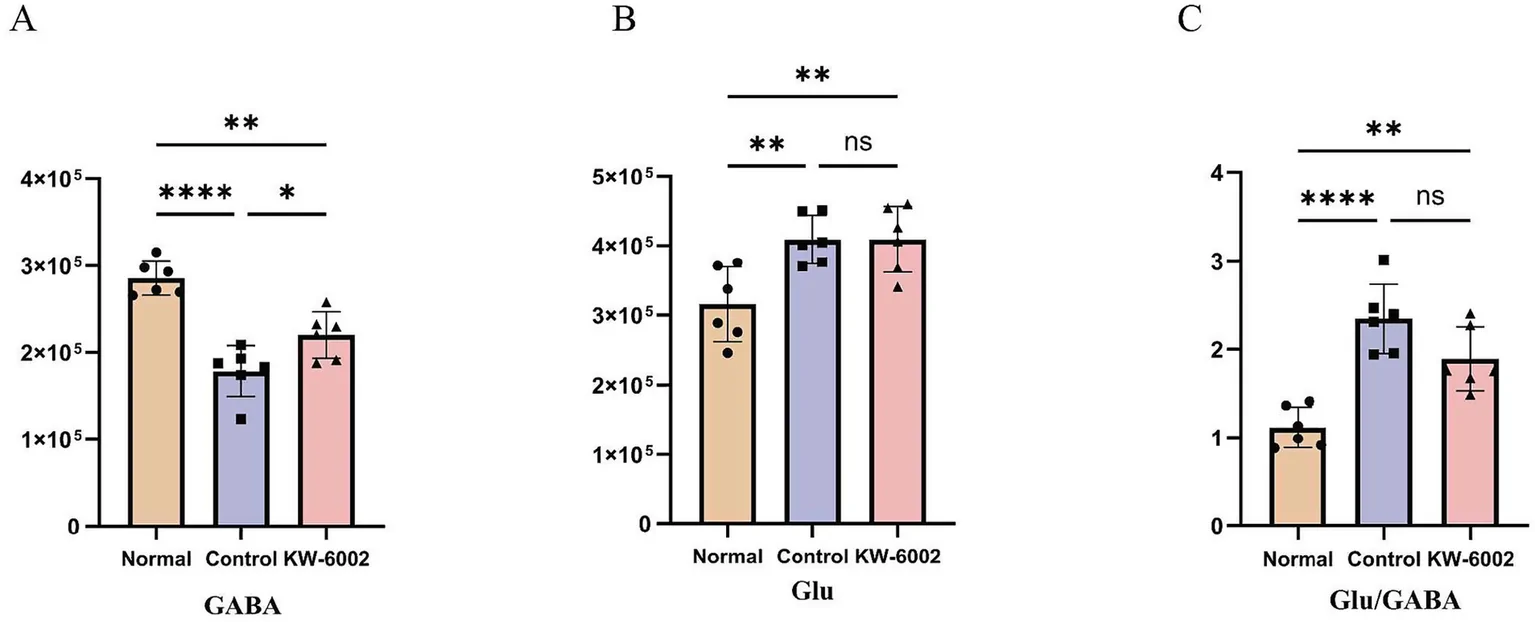
Targeted metabolomic quantification of neurotransmitters in the mouse cerebral cortex. **(A)** GABA content in the cerebral cortex; **(B)** Glu content in the cerebral cortex; **(C)** The Glu/GABA ratio. ^*^p < 0.05; ^**^p < 0.01; ^****^p < 0.0001 indicate statistical significance between groups; ns (*p* > 0.05) indicates no significant difference between groups. All data are presented as mean ± SD (*n* = 6).

### 3.3 KW-6002 modulates the A2AR-coupled Gs protein signaling pathway

To further investigate the underlying causes of the imbalance between excitatory and inhibitory neurotransmission in the mouse brain, the GABA and Glu contents in the cerebral cortex, hippocampus, and striatum were quantified using ELISA. The results showed that 5 mg/kg of KW-6002 effectively reversed the decrease in GABA levels in the cortex, hippocampus, and striatum (Figures 3A–C). However, no significant effects were observed on Glu levels in the cerebral cortex and striatum, whereas a reduction in hippocampal Glu levels was noted compared to the Control group (Figures 3D–F).

**Figure 3:**
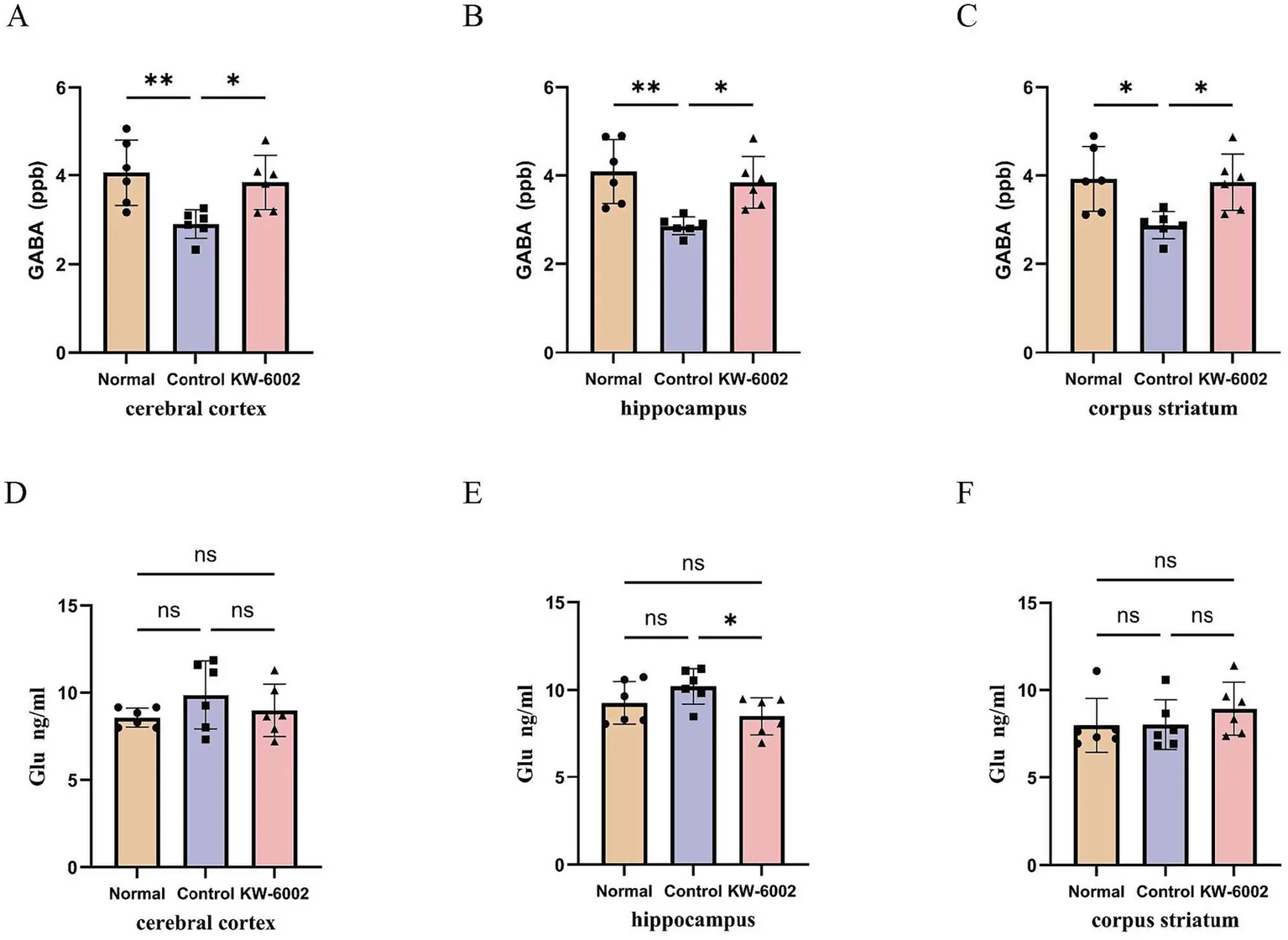
Regional distribution of GABA and Glu levels in the mouse cortex, hippocampus, and striatum. GABA concentrations in the cerebral cortex **(A)**, hippocampus **(B)**, and striatum **(C)**. Glu concentrations in the cerebral cortex **(D)**, hippocampus **(E)**, and striatum **(F)**. Data are presented as mean ± SD (*n* = 6). ^*^p < 0.05; ^**^p < 0.01 indicate statistically significant differences between groups; ns indicates no significant difference (*p* > 0.05).

KW-6002 treatment significantly attenuated the elevations of cAMP, PKA, and p-CREB in the mouse striatum induced by HBO exposure (Figures 4A–C). Furthermore, Western blot analysis revealed that while HBO exposure alone did not significantly alter AMPAR phosphorylation levels in the striatum, KW-6002 administration markedly reduced the phosphorylation state of AMPAR. Concurrently, KW-6002 restored the phosphorylation levels of GABAR, which were diminished following HBO exposure (Figures 4D–F).

**Figure 4:**
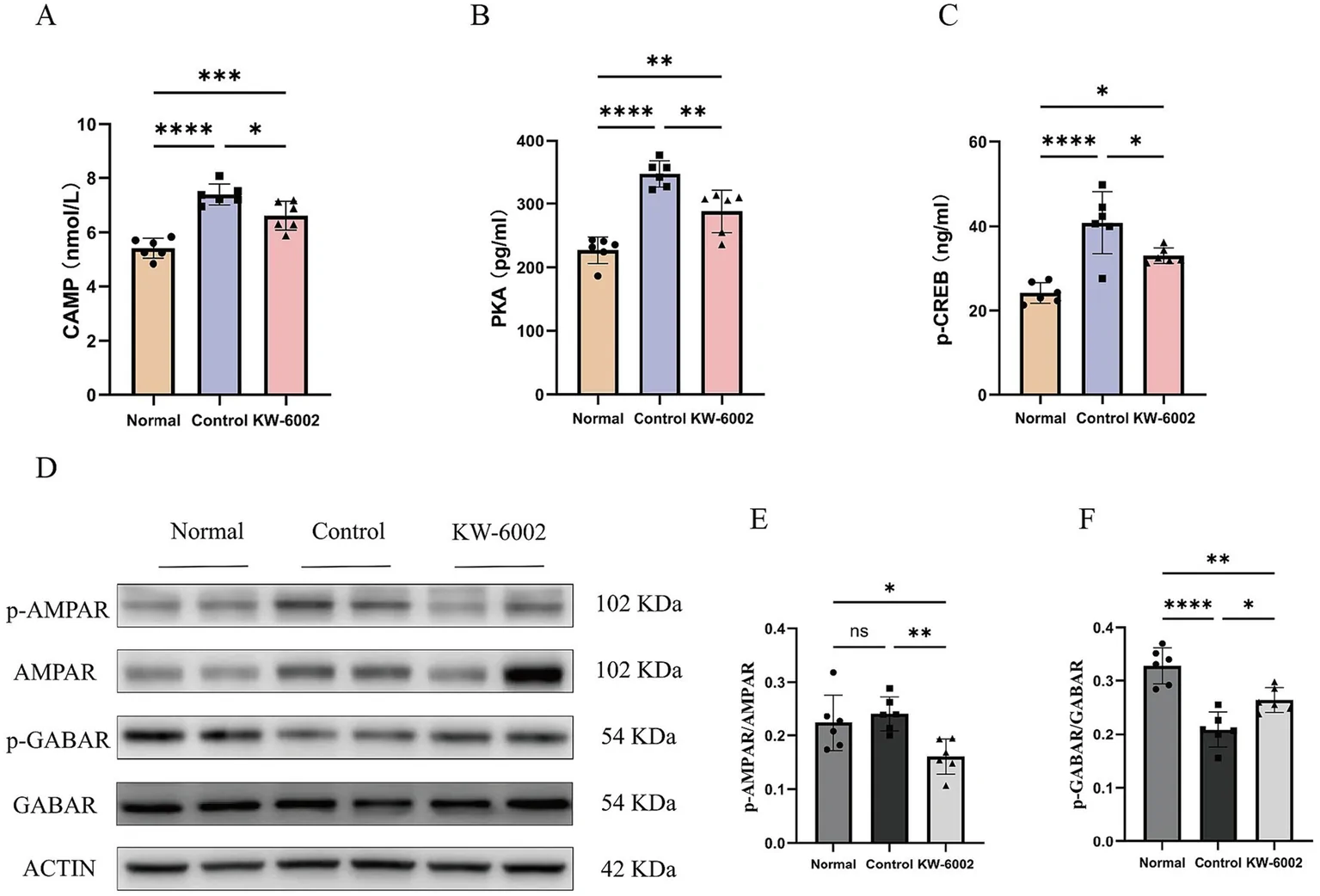
Effects of KW-6002 on the A2AR-coupled Gs protein signaling pathway. Levels of **(A)** cAMP, **(B)** PKA, and **(C)** p-CREB in the mouse striatum. **(D)** Representative Western blot bands showing the total and phosphorylated protein expression of AMPAR and GABAR, along with the statistical quantification of phosphorylation levels for AMPAR **(E)** and GABAR **(F)**. ^*^p < 0.05; ^**^p < 0.01; ^***^p < 0.001; ^****^p < 0.0001 indicate statistically significant differences between groups; ns indicates no significant difference (*p* > 0.05). Data are presented as mean ± SD (*n* = 6).

### 3.4 KW-6002 attenuates HBO-induced oxidative stress in the cerebral cortex

HBO exposure led to a significant elevation of MDA levels in the mouse cerebral cortex. Compared with Control group, KW-6002 markedly reduced MDA content to levels comparable to those of Normal group (Figure 5A). Furthermore, KW-6002 effectively reversed the decline in CAT activity induced by HBO exposure (Figure 5B). Regarding GPx, its activity decreased following HBO exposure and was further inhibited after KW-6002 administration (Figure 5C).

**Figure 5:**
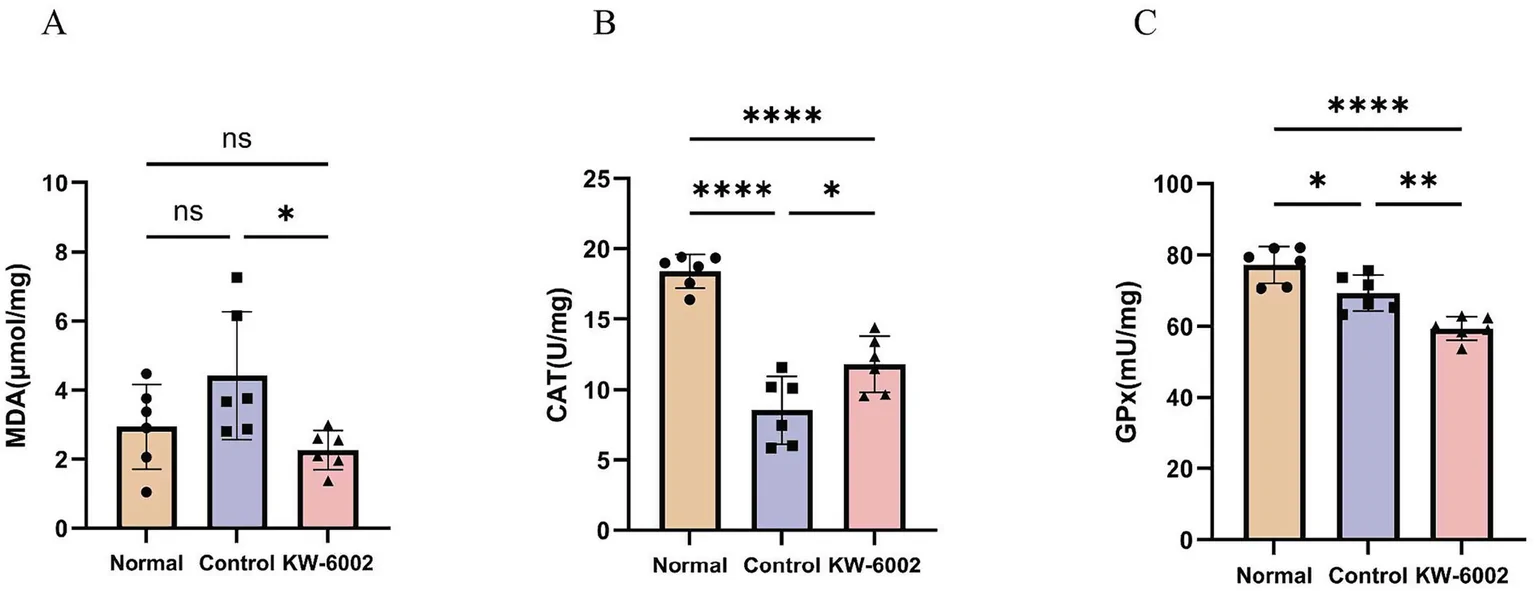
Oxidative stress levels in the mouse cerebral cortex. **(A)** MDA content, **(B)** CAT activity, and **(C)** GPx activity in the cerebral cortex. ^*^p < 0.05; ^**^p < 0.01; ^****^p < 0.0001 indicate statistically significant differences between groups; ns indicates no significant difference (*p* > 0.05). Data are presented as mean ± SD (*n* = 6).

### 3.5 KW-6002 attenuates neuroinflammation in the mouse striatum

The mRNA expression levels of pro-inflammatory cytokines, including IL-1β, IL-6, and TNF-α, were quantified in the striatum using RT-qPCR. The results demonstrated that the mRNA levels of all three neuroinflammatory cytokines were significantly elevated in the Control group. Notably, the 5 mg/kg KW-6002 group effectively reversed this trend (Figure 6).

**Figure 6:**
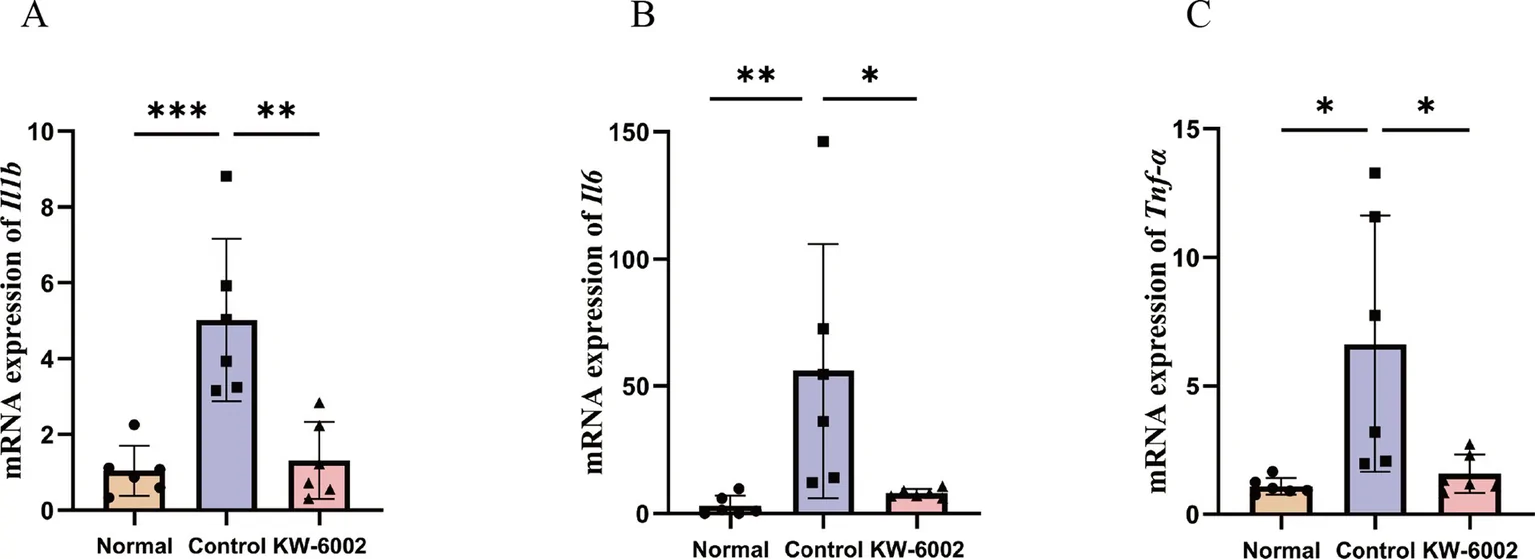
mRNA expression levels of neuroinflammatory cytokines in the mouse striatum. **(A)** *IL-1β* mRNA levels; **(B)** *IL-6* mRNA levels; **(C)** *TNF-α* mRNA levels. ^*^p < 0.05; ^**^p < 0.01; ^***^p < 0.001, indicate statistically significant differences between groups. Data are presented as mean ± SD (*n* = 6).

## 4 Discussion

The pathogenesis of CNS-OT is highly complex and currently considered to involve multiple interrelated factors. These include the toxic effects of excessive reactive oxygen species (ROS) overproduced during HBO exposure, the imbalance between excitatory and inhibitory neurotransmitters, pressure-induced alterations in cellular receptor functions, as well as acidosis and changes in cerebral blood flow (Groborz et al., 2025). Studies have indicated that substances such as ammonia (NH_3_) (Faiman et al., 1977), nitric oxide (NO) (Sato et al., 2001; Demchenko and Piantadosi, 2006), and adenosine (You et al., 2025; Chen et al., 2014; Xie et al., 2017) participate in the onset and development of CNS-OT by modulating neurological functions. Currently reported pharmacological candidates for counteracting CNS-OT primarily include antioxidants, pyridoxine, and low-dose sedatives/anesthetics. However, only a few antioxidants, such as disulfiram, glutathione, and caffeine, have been confirmed to prevent or alleviate CNS-OT (Groborz et al., 2025; Sato et al., 2001); the efficacy of most potent antioxidants, such as Vitamin E (Xiao et al., 2012), N-acetylcysteine (Xiao et al., 2012), allopurinol (Hoppe et al., 1984), hypoxanthine (Hoppe et al., 1984), and superoxide dismutase (Manning, 2016), remains inconclusive. Additionally, sedative-hypnotics, anticonvulsants, and anesthetics—such as phenobarbital (Mayevsky and Shaya, 1980; Lembeck and Beubler, 1977), diazepam and other benzodiazepines (Mayevsky and Shaya, 1980; Lembeck and Beubler, 1977), chloroform (Bean, 1945), baclofen (Lembeck and Beubler, 1977), carbamazepine (Reshef et al., 1991), chlorpromazine (Manning, 2016), excitatory amino acid antagonists (Colton and Colton, 1985), vigabatrin (Jung et al., 1977; Gram et al., 1989), and adenosinergic drugs—can delay the latency to HBO-induced seizures (Chen et al., 2016; Ari et al., 2019). Nevertheless, the combination of sedatives or anesthetics with hyperbaric oxygen exerts synergistic inhibitory effects on the respiratory and circulatory systems and may mask prodromal warning symptoms of seizures. Even at low doses, these drugs inevitably impair high-level neurological functions. During diving operations, divers must continuously monitor critical parameters such as depth, duration, and gas supply pressure while responding to a complex and dynamic underwater environment; any decline in cognitive or motor performance significantly elevates the risk of accidents. Consequently, there is widespread concern regarding the safety of sedative use among the diving community (Moon, 2025; Querido et al., 2023).

Under HBO exposure, oxygen tension in brain tissues increase substantially, leading to a sharp enhancement of aerobic oxidative processes in neurons. As the most critical substrate for the synthesis of ATP—the end product of aerobic oxidation—adenosine levels are significantly affected. Consequently, adenosine directly participates in modulating various physiological effects of HBO and is closely associated with the onset of oxygen-induced seizures. Adenosine A2AR are widely expressed in the nervous system, particularly in the basal ganglia, where they co-regulate neuronal excitability in conjunction with dopamine D2R (Schiffmann et al., 2007). A2AR are markedly enriched in the striatum, whereas a knockout-validated mouse binding study found substantially lower receptor binding in the cortex (Jarvis and Williams, 1989; Schiffmann et al., 1991; Dixon et al., 1996; Rosin et al., 1998; Lopes et al., 2004). We therefore prioritized the striatum for exploratory analyses of signaling markers potentially associated with A2A receptor antagonism and assessed pro-inflammatory cytokine mRNA in the same region. The cortex was selected for targeted metabolomic and oxidative-stress analyses because free-radical formation and lipid peroxidation have previously been demonstrated in cortical tissue under high oxygen pressure or after HBO exposure (Dirks and Faiman, 1982; Chavko and Harabin, 1996; Ay et al., 2007). Recent studies have indicated a close relationship between A2AR and excitatory brain injury. Bruzzese et al. found that exposing rats to 2 ATA HBO for 4 h resulted in decreased blood adenosine levels and downregulated A2AR expression in brain tissues, which was speculated to be an adaptive response. A2AR deficiency can induce vasoconstriction and a subsequent reduction in cerebral blood flow, thereby decreasing cerebral oxygen delivery; this represents an endogenous protective mechanism against CNS-OT (Boussuges et al., 2022). Furthermore, Li et al. demonstrated in a transient focal cerebral ischemia model that A2AR gene deletion inhibited glutamate efflux during both the ischemic and reperfusion phases and ameliorated brain injury, suggesting that the protective effect of A2AR inactivation against ischemic brain damage is related to the inhibition of glutamate-dependent toxicity (Gui et al., 2009). Other studies have shown that A2AR on neuronal membranes are closely linked to the stability of GABAR; specifically, blocking A2AR can reduce the decay of GABAAR currents and improve receptor stability (Sebastião and Ribeiro, 2023). Additionally, research has found that A2AR activation can enhance NMDA receptor (NMDAR) function via a PKA-dependent pathway, promoting glutamate release while inhibiting the activity of GABAergic interneurons (Popoli et al., 1995; Shen et al., 2013). Based on the aforementioned findings, we hypothesize that adenosine A2AR likely plays a pivotal role in HBO-induced CNS-OT, warranting further in-depth exploration.

Studies have shown that the imbalance of neuronal excitatory/inhibitory (E/I) is a key factor in neuronal hyperexcitability during CNS-OT (Groborz et al., 2025). HBO reduces GABA levels in the brain, and adenosine A2AR can integrate GABA, dopamine, and Glu signaling pathways, thereby achieving fine-tuned regulation of striatal neural activity (Yu et al., 2024; Cabello et al., 2009). When GABA content in rat brain tissue decreases by 30–40% from the basal level, oxygen-induced seizures can occur. The use of GABA transporter blockers such as tiagabine and SNAP5114, as well as the GABA transaminase inhibitor vigabatrin, can reduce the decline of GABA content in the striatum, or even elevate GABA content to sufficient levels to exert a preventive effect against CNS-OT (Alekseeva et al., 2023; Demchenko et al., 2021). In the present study, the results of targeted metabolomics in the mouse cerebral cortex also indicated that after hyperbaric oxygen exposure, GABA content in the Control group significantly decreased, while the content of the excitatory neurotransmitter Glu significantly elevated, indicating an imbalance in the ratio of excitatory to inhibitory (E/I) neurotransmitters in the cerebral cortex. Following KW-6002 administration, GABA content significantly recovered, whereas Glu levels showed no significant change. This indicates that KW-6002 primarily counteracts oxygen-induced seizures by maintaining GABA content at a relatively high level.

The functions of excitatory receptors, such as AMPAR and NMDAR, as well as inhibitory GABA receptors (primarily GABAAR), are closely related to their phosphorylation status. Phosphorylation and dephosphorylation serve as core mechanisms regulating channel properties, membrane localization, endocytosis/recycling, and the efficiency, strength, and plasticity of synaptic transmission. Our study found that HBO exposure significantly decreased the phosphorylation levels of GABAR; although the phosphorylation levels of the glutamatergic ionotropic AMPAR showed no statistical difference compared to the Control group, they exhibited an upward trend. This suggests that the activities of Glu receptors and GABA receptors are indeed altered during the pathogenesis of CNS-OT; specifically, the alteration in GABA receptor activity may be a critical link exacerbating E/I imbalance. Simultaneously, our results showed that KW-6002 significantly decreased the phosphorylation levels of the GluA1 subunit of AMPAR at Ser845 and increased the phosphorylation levels of GABAA R, suggesting that the effective modulation of receptor activities is an important pathway for its antagonism against oxygen-induced seizures. Studies have shown a close link between adenosine A2AR and AMPAR. In models of cerebral hypoxia/reperfusion injury, increased membrane expression of A2AR stimulates the membrane expression of calcium-permeable AMPAR, mediating enhanced synaptic transmission and neuronal damage (Qin et al., 2021). Increased phosphorylation at the Ser845 site of the AMPAR subunit GluA1 reduces receptor endocytosis and increases GluA1 expression on the neuronal membrane (Sathler et al., 2020). Elevated extracellular adenosine can upregulate membrane A2AR levels, indirectly enhancing synaptic transmission and leading to increased presynaptic glutamate release and enhanced postsynaptic AMPAR function (Stockwell et al., 2017; Cunha, 2005). This may be related to the concentration of A2ARs in GABAergic medium spiny neurons (MSNs) of the striatum, nucleus accumbens, olfactory tubercle, and other dopamine-rich brain regions (de Lera Ruiz et al., 2014). Regarding the correlation between A2AR and GABAR, research has shown that neurosteroids can increase the phosphorylation of the GABAAR β3 subunit at the Ser408/409 sites, thereby exerting anxiolytic and anticonvulsant effects (Vien et al., 2022). PKA can phosphorylate the β3 subunit (McDonald et al., 1998), but Ser408/409 phosphorylation is not regulated exclusively by PKA. Biochemical studies identified PKC as another kinase capable of phosphorylating this region (McDonald and Moss, 1997), and activation of either PKA or PKC increased β3 Ser408/409 phosphorylation in cultured striatal neurons (Brandon et al., 2003). In cultured cortical neurons, basal β3 phosphorylation was predominantly PKC-dependent (Brandon et al., 2000). Moreover, neuronal studies have demonstrated an initial PKC-associated increase in β3 phosphorylation followed by PP2A-mediated dephosphorylation, illustrating that the phosphorylation measured at a given time reflects the balance of several regulatory processes (Jovanovic et al., 2004). This regulation may be relevant to inhibitory transmission: phosphorylation within the β3 Ser408/409-containing motif reduces binding of the endocytic adaptor AP2 (Kittler et al., 2005), while an experimental status epilepticus study linked reduced PKC-associated Ser408/409 phosphorylation to receptor internalization and impaired synaptic inhibition (Terunuma et al., 2008). Nevertheless, the functional effect of β3 phosphorylation depends on the experimental context (McDonald et al., 1998; Brandon et al., 2003). Although A2AR activation can stimulate adenylyl cyclase and cAMP-related signaling (Kwon and Koh, 2020), the increased phosphorylated β3 Ser408/409-to-total β3 ratio observed after KW-6002 treatment is therefore not necessarily inconsistent with a reduction in A2AR-associated cAMP signaling. In the mouse model of oxygen-induced seizures caused by HBO exposure, we confirmed that the activities of both excitatory AMPARs and inhibitory GABA receptors undergo significant changes during the disease process, which may be closely related to the enhanced activity of A2AR on the neuronal membrane. As an A2AR antagonist, KW-6002 can effectively inhibit AMPAR activity while simultaneously enhancing GABAR activity, which is inseparable from its effect against oxygen-induced seizures. Investigating the mechanisms of CNS-OT from the perspective of excitatory/inhibitory receptor phosphorylation provides direct molecular evidence for understanding the pathological process of oxygen-induced seizures and offers potential targets for the development of interventional strategies.

To further elucidate the pivotal role of A2AR signaling pathway in the pathogenesis of oxygen-induced seizures, we conducted a detailed investigation of this cascade. Previous research has demonstrated that the activation of adenosine A2AR triggers cyclase (AC) through Gs protein coupling, which subsequently catalyzes the conversion of ATP into the second messenger cyclic adenosine monophosphate (cAMP). Elevated cAMP levels then activate protein kinase A (PKA), which regulates neuronal excitability, synaptic transmission, and plasticity by phosphorylating various downstream target proteins (Borea et al., 2018; Wu et al., 2018). Our findings demonstrate that hyperbaric oxygen (HBO) exposure significantly upregulates the expression levels of cAMP, PKA, and p-CREB, whereas KW-6002 effectively suppresses this upregulation. As an A2AR antagonist, KW-6002 exerts its protective effects through a dual regulatory mechanism: first, it reduces AMPAR phosphorylation by inhibiting A2AR-mediated signaling, thereby decreasing synaptic excitability; second, it enhances central inhibition by promoting GABA levels and activating GABAAR phosphorylation. This dual modulation enables the maintenance of a relatively stable neurone during HBO exposure. Consequently, the present study preliminarily indicates that KW-6002 exerts neuroprotective effects across multiple brain regions by antagonizing the overactivation of the A2AR-mediated cAMP/PKA/CREB signaling pathway and restoring inhibitory GABAergic neurotransmission.

Studies have indicated that A2AR are closely related to oxidative stress. Chronic A2AR stimulation can exacerbate oxidative stress (Persson et al., 2015), whereas blocking A2AR can prevent the accumulation of ROS (Aires et al., 2019) and reduce oxidative stress levels in endothelial cells (ECs) (Thakur et al., 2010). HBO exposure significantly increased oxidative stress levels, as evidenced by elevated levels of the lipid peroxidation product MDA, indicating oxidative damage to cell membrane structures (Leveque et al., 2023). The decrease in CAT activity reflects the massive consumption of this key ROS-scavenging enzyme under high oxidative load conditions. However, the antioxidant enzyme GPx may become inactivated due to severe oxidative damage or the depletion of its substrate, glutathione (GSH). Our findings revealed marked alterations in oxidative stress markers following HBO exposure, characterized by increased MDA content and significantly reduced CAT and GPx activities. Following intervention with KW-6002, MDA levels significantly decreased and CAT activity was partially restored; however, GPx activity was further reduced. The reduction in MDA levels and the elevation in CAT activity suggest that KW-6002 possesses the capacity to attenuate oxidative damage. Conversely, the further decline in GPx activity suggests a possible inhibitory effect of KW-6002 on this specific enzyme. This observation is supported by Yeung et al. (Yeung et al., 2025), who found that after administration of KW-6002, *gpx1* expression was reduced to 0.19 times that of the Control group, indicating that *gpx1* is regulated by the A2A signaling pathway and confirming a positive regulatory relationship between the A2AR pathway and GPx1. It is important to note that oxidative stress is a dynamic equilibrium process; differences in experimental models, pressures, and exposure durations may lead to inconsistent or even contradictory results in oxidative biomarkers (Leveque et al., 2023; De Wolde et al., 2021).

Silke et al. (De Wolde et al., 2021) summarized the effects of HBOT on various physiological parameters, suggesting that neuroinflammation, alongside oxidative stress, represents a crucial component of HBO treatment. Neuroinflammation encompasses a broad spectrum of biological and cellular responses of the nervous system to injury, infection, and neurodegenerative diseases, primarily driven by the activation of neuroimmune cells such as astrocytes and microglia (Kwon and Koh, 2020). Common hallmarks of neuroinflammation include the aberrant synthesis of pro-inflammatory cytokines, such as TNF-α and IL-1β, as well as elevated levels of reactive oxygen species (ROS) and reactive nitrogen species (Stöberl et al., 2023). Adenosine A2AR are highly correlated with inflammatory factors; inhibiting A2AR has been shown to attenuate inflammatory responses induced by pro-inflammatory cytokines (Marti Navia et al., 2020). Furthermore, A2AR antagonists have demonstrated anti-inflammatory effects in numerous CNS disorders, an effect secondary to their inhibition of glutamate efflux and the resulting excitotoxicity (Chen et al., 2007; Chen and Pedata, 2008). Our study found that during the pathogenesis of CNS-OT, the mRNA expression levels of IL-1β, TNF-α, and IL-6 were significantly upregulated. KW-6002 significantly reduced the mRNA levels of these inflammatory cytokines, indicating that the occurrence of oxygen-induced seizures and their subsequent effects may be closely related to neuroinflammatory cytokines. Similarly, the protective effect of adenosine A2AR against oxygen-induced seizures may be intimately linked to its anti-inflammatory properties. Currently, no systematic research has confirmed whether neuroinflammation serves as a primary trigger or a core feature resulting from the seizures in CNS-OT. Exposure to high concentrations of oxygen may induce neuronal damage via oxidative stress, making inflammatory responses potentially secondary, transient, and non-essential events, yet they likely contribute to subsequent sequelae. Domachevsky et al. found that mice exhibited transient cognitive impairment following oxygen-induced seizures. Specifically, impairments in spatial memory and non-spatial learning and memory appeared early—potentially related to cytotoxic edema—and recovered by 30 days post-seizure. In contrast, impairments in visual/recognition memory and anxiety-like behaviors appeared later, possibly linked to blood–brain barrier disruption and secondary to the inflammatory response following the primary seizure injury; notably, anxiety-like behaviors persisted 30 days post-seizure (Domachevsky et al., 2013). Their research further revealed that apoptotic levels in hippocampal tissue remained significantly higher than those in the Control group one week after the seizure (Domachevsky et al., 2012). These findings suggest that a more comprehensive and systematic understanding of the impact of CNS-OT on the body is required, along with a full consideration of the protective role of preventive measures. The fact that KW-6002 can significantly inhibit the mRNA elevation of inflammatory cytokine levels indicates that it can provide more comprehensive protection against CNS-OT.

In conclusion, this study evaluated istradefylline as a prophylactic intervention administered 1 h before HBO exposure. Our findings suggest that pretreatment may protect against HBO-induced CNS oxygen toxicity seizures. The underlying mechanisms involve multiple targets and signaling pathways, including the modulation of neurotransmitter levels and their receptor activities by inhibiting the overactivation of the cAMP/PKA/CREB pathway, as well as the alleviation of oxidative stress and the suppression of neuroinflammation. Given that istradefylline is an established, marketed drug with a proven human safety profile, it holds significant potential for clinical application in counteracting CNS-OT in humans, presenting promising prospects for further development. Furthermore, our findings are consistent with a potential role for A2AR-related signaling in HBO-induced CNS-OT, but they do not establish that istradefylline’s protective effects are specifically mediated by A2AR. Genetic and complementary pharmacological studies are needed to test this receptor dependence. Conducting research on the pathogenic mechanisms and pharmacological interventions of CNS-OT targeting _A2AR_ can provide novel insights for a comprehensive and systematic understanding of the disease etiology. This approach addresses the current lack of active preventive measures, ultimately providing a robust guarantee for the more extensive and safe utilization of HBO.

In the present study, we preliminarily explored the effects and underlying mechanisms of istradefylline in the prevention and treatment of CNS-OT, yielding several beneficial new findings; however, numerous questions warrant further investigation. Specifically regarding the mechanism of action of istradefylline, it is necessary to integrate the mechanisms of HBO and the pathogenesis of epilepsy to explore—from multiple perspectives—the downstream pathways and targets affected by istradefylline following its inhibition of the cAMP/PKA/CREB signaling cascade. This includes its effects on dopamine D2R and their regulated motor circuits, its regulatory role in cerebrovascular function and cerebral blood flow, and the assessment of additional indicators related to oxidative damage, thereby providing more experimental evidence to confirm the prophylactic and therapeutic effects of istradefylline. Furthermore, based on our finding that istradefylline significantly inhibits the elevation of inflammatory cytokine expression during oxygen-induced seizures, we believe that its impact on the post-seizure sequelae could be further explored. For instance, investigating its effect on postictal cognitive decline would facilitate a more systematic understanding of the impact of oxygen-induced seizures on the body and the comprehensive protective role of istradefylline. The mechanistic analyses included only six mice per group, which may limit the precision of the findings and the ability to detect smaller effects. Because only male mice were studied, their applicability to females remains unknown. Larger studies including both sexes are needed to confirm these findings. Exposure-run effects were not modeled; future studies should account for them statistically and consider separating mice during HBO exposure. Regarding the eventual development of istradefylline as a clinical medication for the prevention and treatment of CNS-OT, since exposure to high pressure and HBO exerts certain effects on the functions of various organs, it remains necessary to systematically study the pharmacokinetics and safety profiles of istradefylline under hyperbaric conditions.

## Data Availability

The raw data supporting the conclusions of this article will be made available by the authors, without undue reservation.

## References

[ref1] Aires I. D. Boia R. Rodrigues-Neves A. C. Madeira M. H. Marques C. Ambrósio A. F. . (2019). Blockade of microglial adenosine A2A receptor suppresses elevated pressure-induced inflammation, oxidative stress, and cell death in retinal cells. Glia 60, 526–534. doi: 10.1002/glia.23579, 30667095 PMC6590475

[ref2] Alekseeva O. S. Gerda B. A. Zhilyaeva A. S. Demchenko I. T. (2023). Anticonvulsant efficacy of inhibition of synaptic and extrasynaptic GABA-transporters in the prevention of hyperbaric oxygen seizures. J. Evol. Biochem. Physiol. 59, 709–718. doi: 10.1134/s0022093023030055

[ref3] Ari C. Koutnik A. P. DeBlasi J. Landon C. Rogers C. Q. Vallas J. . (2019). Delaying latency to hyperbaric oxygen-induced CNS oxygen toxicity seizures by combinations of exogenous ketone supplements. Physiol. Rep. 7:e13961. doi: 10.14814/phy2.13961 PMC6317287 30604923

[ref4] Ay H. Topal T. Özler M. Uysal B. Korkmaz A. Öter S. . (2007). Persistence of hyperbaric oxygen-induced oxidative effects after exposure in rat brain cortex tissue. Life Sci. 80, 2025–2029. doi: 10.1016/j.lfs.2007.03.002, 17407783

[ref5] Bean J. W. (1945). Effects of oxygen at increased pressure. Physiol. Rev. 25, 1–147.

[ref6] Borea P. A. Gessi S. Merighi S. Vincenzi F. Varani K. (2018). Pharmacology of adenosine receptors: the state of the art. Physiol. Rev. 98, 1591–1625. doi: 10.1152/physrev.00049.2017, 29848236

[ref7] Boussuges A. Bourenne J. Eloufir F. Fromonot J. Mottola G. Risso J. J. . (2022). Contribution of adenosine in the physiological changes and injuries secondary to exposure to extreme oxygen pressure in healthy subjects. Biomedicine 10:2059. doi: 10.3390/biomedicines10092059, 36140160 PMC9495509

[ref8] Brandon N. J. Delmas P. Kittler J. T. McDonald B. J. Sieghart W. Brown D. A. . (2000). GABA (A) receptor phosphorylation and functional modulation in cortical neurons by a protein kinase C-dependent pathway. J. Biol. Chem. 275, 38856–38862. doi: 10.1074/jbc.m004910200 10978327

[ref9] Brandon N. J. Jovanovic J. N. Colledge M. Kittler J. T. Brandon J. M. Scott J. D. . (2003). A-kinase anchoring protein 79/150 facilitates the phosphorylation of GABAA receptors by cAMP-dependent protein kinase via selective interaction with receptor β subunits. Mol. Cell. Neurosci. 22, 87–97. doi: 10.1016/s1044-7431(02)00017-9 12595241

[ref10] Cabello N. Gandía J. Bertarelli D. C. G. Watanabe M. Lluís C. Franco R. . (2009). Metabotropic glutamate type 5, dopamine D 2 and adenosine A 2a receptors form higher-order oligomers in living cells. J. Neurochem. 109, 1497–1507. doi: 10.1111/j.1471-4159.2009.06078.x, 19344374 PMC3925975

[ref11] Chavko M. Harabin A. L. (1996). Regional lipid peroxidation and protein oxidation in rat brain after hyperbaric oxygen exposure. Free Radical Bio Med. 20, 973–978. doi: 10.1016/0891-5849(95)02181-7, 8743983

[ref12] Chen J.-F. Cunha R. A. (2020). The belated US FDA approval of the adenosine A2A receptor antagonist istradefylline for treatment of Parkinson’s disease. Purinergic Signalling. 16, 167–174. doi: 10.1007/s11302-020-09694-2, 32236790 PMC7367999

[ref13] Chen J.-F. Pedata F. (2008). Modulation of ischemic brain injury and Neuroinflammation by adenosine A2A receptors. CPD. 14, 1490–1499. doi: 10.2174/138161208784480126, 18537672

[ref14] Chen J.-F. Sonsalla P. K. Pedata F. Melani A. Domenici M. R. Popoli P. . (2007). Adenosine A2A receptors and brain injury: broad spectrum of neuroprotection, multifaceted actions and “fine tuning” modulation. Prog. Neurobiol. 83, 310–331. doi: 10.1016/j.pneurobio.2007.09.002, 18023959

[ref15] Chen Y.-l. Zhang Y.-n. Wang Z.-z. Xu W.-g. Li R.-p. Zhang J.-d. (2014). Studies on the role of adenosine in central nervous system oxygen toxicity induced by hyperbaric oxygen. Chin. J. Naut. Med. Hyperbaric Med. 21, 153–156.

[ref16] Chen Y.-l. Zhang Y.-n. Wang Z.-z. Xu W.-g. Li R.-p. Zhang J.-d. (2016). Effects of adenosine metabolism in astrocytes on central nervous system oxygen toxicity. Brain Res. 1635, 180–189. doi: 10.1016/j.brainres.2016.01.026 26806404

[ref17] Ciarlone G. E. Hinojo C. M. Stavitzski N. M. Dean J. B. (2019). CNS function and dysfunction during exposure to hyperbaric oxygen in operational and clinical settings. Redox Biol. 27:101159. doi: 10.1016/j.redox.2019.101159, 30902504 PMC6859559

[ref18] Colton C. A. Colton J. S. (1985). Blockade of hyperbaric oxygen induced seizures by excitatory amino acid antagonists. CAN J PHYSIOL PHARM. 63, 519–521. doi: 10.1139/y85-090, 2864124

[ref19] Cunha R. A. (2005). Neuroprotection by adenosine in the brain: from A (1) receptor activation to A (2A) receptor blockade. Purinergic Signal 1, 111–134. doi: 10.1007/s11302-005-0649-1, 18404497 PMC2096528

[ref20] Đambić V. Mihaljević Z. Drenjančević I. Jukić I. Šušnjara P. Kibel A. (2025). The role of adenosine A1 and A2a receptors in cerebral blood vessel reactivity of Sprague Dawley rats exposed to hyperbaric oxygenation. Molecules 30:2918. doi: 10.3390/molecules30142918, 40733187 PMC12298982

[ref21] de Lera Ruiz M. Lim Y.-H. Zheng J. (2014). Adenosine A2A receptor as a drug discovery target. J. Med. Chem. 57, 3623–3650. doi: 10.1021/jm4011669, 24164628

[ref22] De Wolde S. D. Hulskes R. H. Weenink R. P. Hollmann M. W. Van Hulst R. A. (2021). The effects of hyperbaric oxygenation on oxidative stress, inflammation and angiogenesis. BIOMOLECULES. 11:1210. doi: 10.3390/biom11081210, 34439876 PMC8394403

[ref23] Demchenko I. T. Piantadosi C. A. (2006). Nitric oxide amplifies the excitatory to inhibitory neurotransmitter imbalance accelerating oxygen seizures. Undersea Hyperb. Med. 33, 169–174. 16869530

[ref24] Demchenko I. T. Zhilyaev S. Y. Platonova T. F. Alekseeva O. S. Nikitina E. R. (2021). Inhibition of GABA-transaminase and GABA-transporters in the brain by vigabatrin and tiagabine prevents seizure development in rats breathing hyperbaric oxygen. J. Evol. Biochem. Physiol. 57, 1101–1109. doi: 10.1134/s0022093021050112

[ref25] Dirks R. C. Faiman M. D. (1982). Free radical formation and lipid peroxidation in rat and mouse cerebral cortex slices exposed to high oxygen pressure. Brain Res. 248, 355–360. doi: 10.1016/0006-8993(82)90594-7 7139281

[ref26] Dixon A. K. Gubitz A. K. Sirinathsinghji D. J. S. Richardson P. J. Freeman T. C. (1996). Tissue distribution of adenosine receptor mRNAs in the rat. Br. J. Pharmacol. 118, 1461–1468. doi: 10.1111/j.1476-5381.1996.tb15561.x, 8832073 PMC1909676

[ref27] Domachevsky L. Pick C. G. Arieli Y. Krinsky N. Abramovich A. Eynan M. (2012). Do hyperbaric oxygen-induced seizures cause brain damage? Epilepsy Res. 100, 37–41. doi: 10.1016/j.eplepsyres.2012.01.004, 22293507

[ref28] Domachevsky L. Rachmany L. Barak Y. Rubovitch V. Abramovich A. Pick C. G. (2013). Hyperbaric oxygen-induced seizures cause a transient decrement in cognitive function. Neuroscience 247, 328–334. doi: 10.1016/j.neuroscience.2013.05.052, 23732232

[ref29] Faiman M. D. Nolan R. J. Baxter C. F. Dodd D. E. (1977). Brain γ-aminobutyric acid, glutamic acid decarboxylase, glutamate, and ammonia in mice during hyperbaric oxygenation. J. Neurochem. 28, 861–865. doi: 10.1111/j.1471-4159.1977.tb10640.x, 894292

[ref30] Ferré S. Bonaventura J. Zhu W. Hatcher-Solis C. Taura J. Quiroz C. . (2018). Essential control of the function of the Striatopallidal neuron by pre-coupled complexes of adenosine A2A-dopamine D2 receptor Heterotetramers and adenylyl cyclase. Front. Pharmacol. 9:243. doi: 10.3389/fphar.2018.00243, 29686613 PMC5900444

[ref31] Fuxe K. Marcellino D. Borroto-Escuela D. O. Guescini M. Fernández-Dueñas V. Tanganelli S. . (2010). Adenosine–dopamine interactions in the pathophysiology and treatment of CNS disorders. CNS Neurosci. Ther. 16, e18–e42. doi: 10.1111/j.1755-5949.2009.00126.x PMC6493824 20345970

[ref32] Gao Z. (2023). Adenosine A2a receptor and glia. Int. Rev. Neurobiol. 170, 29–48. doi: 10.1016/bs.irn.2023.08.002, 37741695

[ref33] Gram L. Larsson O. M. Johnsen A. Schousboe A. (1989). Experimental studies of the influence of vigabatrin on the GABA system. Br. J. Clin. Pharmacol. 27, 13S–17S. 10.1111/j.1365-2125.1989.tb03455.x PMC1379673 2757904

[ref34] Groborz O. Marsalek P. Sefc L. (2025). New insights into the mechanisms and prevention of central nervous system oxygen toxicity: A prospective review. Life Sci. 360:123169. doi: 10.1016/j.lfs.2024.123169, 39447734

[ref35] Gui L. Duan W. Tian H. Li C. Zhu J. Chen J.-F. . (2009). Adenosine A2A receptor deficiency reduces striatal glutamate outflow and attenuates brain injury induced by transient focal cerebral ischemia in mice. Brain Res. 1297, 185–193. doi: 10.1016/j.brainres.2009.08.050, 19703429

[ref36] Haas H. L. Selbach O. (2000). Functions of neuronal adenosine receptors. N-S Arch Pharmacol. 362, 375–381. doi: 10.1007/s002100000314, 11111831

[ref37] Hoppe S. A. Terrell D. J. Gottlieb S. F. (1984). The effect of allopurinol on oxygen-induced seizures in mice. Aviat. Space Environ. Med. 55, 927–930. 6093764

[ref38] Jarvis M. F. Williams M. (1989). Direct autoradiographic localization of adenosine A2 receptors in the rat brain using the A2-selective agonist, [3H]CGS 21680. Eur. J. Pharmacol. 168, 243–246. doi: 10.1016/0014-2999(89)90571-2, 2558026

[ref39] Jovanovic J. N. Thomas P. Kittler J. T. Smart T. G. Moss S. J. (2004). Brain-derived neurotrophic factor modulates fast synaptic inhibition by regulating GABA A receptor phosphorylation, activity, and cell-surface stability. J. Neurosci. 24, 522–530. doi: 10.1523/JNEUROSCI.3606-03.2004, 14724252 PMC6729993

[ref40] Jung M. J. Lippert B. Metcalf B. W. Böhlen P. Schechter P. J. (1977). γ-VINYL GABA (4-amino-hex-5-enoic acid), A New Selective Irreversible Inhibitor of Gaba-T: Effects on Brain Gaba Metabolism in Mice. J. Neurochem. 29, 797–802. doi: 10.1111/j.1471-4159.1977.tb10721.x, 591956

[ref41] Kittler J. T. Chen G. Honing S. Bogdanov Y. McAinsh K. Arancibia-Carcamo I. L. . (2005). Phospho-dependent binding of the clathrin AP2 adaptor complex to GABA A receptors regulates the efficacy of inhibitory synaptic transmission. Proc. Natl. Acad. Sci. USA 102, 14871–14876. doi: 10.1073/pnas.0506653102, 16192353 PMC1253579

[ref42] Kwon H. S. Koh S.-H. (2020). Neuroinflammation in neurodegenerative disorders: the roles of microglia and astrocytes. Transl. Neurodegener. 9:42. doi: 10.1186/s40035-020-00221-2 PMC7689983 33239064

[ref43] Lembeck F. Beubler E. (1977). Convulsions induced by hyperbaric oxygen: inhibition by phenobarbital, diazepam and baclofen. N-S Arch Pharmacol. 297, 47–51. doi: 10.1007/BF00508809, 870832

[ref44] Leveque C. Mrakic Sposta S. Theunissen S. Germonpré P. Lambrechts K. Vezzoli A. . (2023). Oxidative stress response kinetics after 60 minutes at different (1.4 ATA and 2.5 ATA) hyperbaric hyperoxia exposures. Int. J. Mol. Sci. 24:12361. doi: 10.3390/ijms241512361, 37569737 PMC10418619

[ref45] Lopes L. V. Halldner L. Rebola N. Johansson B. Ledent C. Chen J. F. . (2004). Binding of the prototypical adenosine A2A receptor agonist CGS 21680 to the cerebral cortex of adenosine A1 and A2A receptor knockout mice. Br. J. Pharmacol. 141, 1006–1014. doi: 10.1038/sj.bjp.0705692, 14993095 PMC1574266

[ref46] Manning E. P. (2016). Central nervous system oxygen toxicity and hyperbaric oxygen seizures. Aerosp Med Hum Perf. 87, 477–486. doi: 10.3357/AMHP.4463.2016, 27099087 PMC7092644

[ref47] Marti Navia A. Dal Ben D. Lambertucci C. Spinaci A. Volpini R. Marques-Morgado I. . (2020). Adenosine receptors as neuroinflammation modulators: role of A1agonists and A2Aantagonists. Cells 9:1739. doi: 10.3390/cells9071739, 32708189 PMC7409197

[ref48] Mayevsky A. Shaya B. (1980). Factors affecting the development of hyperbaric oxygen toxicity in the awake rat brain. J. Appl. Physiol. 49, 700–707. doi: 10.1152/jappl.1980.49.4.700, 7440284

[ref49] McCool B. A. Farroni J. S. (2001). A1 adenosine receptors inhibit multiple voltage-gated Ca2+ channel subtypes in acutely isolated rat basolateral amygdala neurons. Br. J. Pharmacol. 132, 879–888. doi: 10.1038/sj.bjp.0703884, 11181429 PMC1572624

[ref50] McDonald B. J. Amato A. Connolly C. N. Benke D. Moss S. J. Smart T. G. (1998). Adjacent phosphorylation sites on GABAA receptor β subunits determine regulation by cAMP-dependent protein kinase. Nat. Neurosci. 1, 23–28. 10.1038/223 10195104

[ref51] McDonald B. J. Moss S. J. (1997). Conserved phosphorylation of the intracellular domains of GABAA receptorβ2 and β3 subunits by cAMP-dependent protein kinase, cGMP-dependent protein kinase, protein kinase C and Ca2+/calmodulin type II-dependent protein kinase. Neuropharmacology 36, 1377–1385. 10.1016/s0028-3908(97)00111-1 9423925

[ref52] Moon RE. Diving precautions and prevention of diving injuries. MSD Manual Professional Edition. 2025 [updated 2025 Jun; cited 2026 June 17]. Available from: https://www.msdmanuals.com/professional/injuries-poisoning/injury-during-diving-or-work-in-compressed-air/diving-precautions-and-prevention-of-diving-injuries

[ref53] Paton D. M. (2020). Istradefylline: adenosine A2A receptor antagonist to reduce "OFF" time in Parkinson's disease. Drugs Today. 56:125. doi: 10.1358/dot.2020.56.2.3098156 32163528

[ref54] Persson P. Friederich-Persson M. Fasching A. Hansell P. Inagi R. Palm F. (2015). Adenosine A2a receptor stimulation prevents proteinuria in diabetic rats by promoting an anti-inflammatory phenotype without affecting oxidative stress. Acta Physiol. 214, 311–318. doi: 10.1111/apha.12511, 25891445

[ref55] Pinna A. Serra M. Morelli M. Simola N. (2018). Role of adenosine A (2A) receptors in motor control: relevance to Parkinson's disease and dyskinesia. J. Neural Transm. (Vienna) 125, 1273–1286. doi: 10.1007/s00702-018-1848-6, 29396609

[ref56] Popoli P. Betto P. Reggio R. Ricciarello G. (1995). Adenosine A2A receptor stimulation enhances striatal extracellular glutamate levels in rats. Eur. J. Pharmacol. 287, 215–217. doi: 10.1016/0014-2999(95)00679-6, 8749040

[ref57] Qin X. Zaki M. G. Chen Z. Jakova E. Ming Z. Cayabyab F. S. (2021). Adenosine Signaling and Clathrin-mediated endocytosis of glutamate AMPA receptors in delayed hypoxic injury in rat hippocampus: role of casein kinase 2. Mol. Neurobiol. 58, 1932–1951. doi: 10.1007/s12035-020-02246-0, 33415682 PMC8018935

[ref58] Querido A. L. Ebbelaar C. F. Wingelaar T. T. (2023). Diving with psychotropic medication: review of the literature and clinical considerations. Diving Hyperb. Med. 53, 259–267. doi: 10.28920/dhm53.3.259-267, 37718301 PMC10735636

[ref59] Reshef A. Bitterman N. Kerem D. (1991). The effect of carbamazepine and ethosuximide on hyperoxic seizures. Epilepsy Res. 8, 117–121. doi: 10.1016/0920-1211(91)90079-U, 2065642

[ref60] Rosin D. L. Robeva A. Woodard R. L. Guyenet P. G. Linden J. (1998). Immunohistochemical localization of adenosine A2A receptors in the rat central nervous system. J. Comp. Neurol. 401, 163–186. doi: 10.1002/(SICI)1096-9861(19981116)401:2<163::AID-CNE2>3.0.CO;2-D, 9822147

[ref61] Salmaso V. Menin S. Moro S. Spalluto G. Federico S. (2025). Adenosine receptors in neuroinflammation and neurodegeneration. Cells 14:1585. doi: 10.3390/cells14201585, 41148800 PMC12563614

[ref62] Sathler M. F. Khatri L. Roberts J. P. Kubrusly R. C. Ziff E. B. Kim S. (2020). Phosphorylation of AMPA Receptor Subunit GluA1 Regulates Clathrin-Mediated Receptor Endocytosis. 1.1 Edn Cold Spring Harbor: Cold Spring Harbor Laboratory Press.

[ref63] Sato T. Takeda Y. Hagioka S. Zhang S. Hirakawa M. (2001). Changes in nitric oxide production and cerebral blood flow before development of hyperbaric oxygen-induced seizures in rats. Brain Res. 918, 131–140. doi: 10.1016/S0006-8993(01)02984-5, 11684051

[ref64] Satyam S. M. El-Tanani M. Alghoul W. I. Abedi M. M. Farook S. F. Alabid I. K. . (2026). Adenosine signaling as a central integrative network in cellular stress responses and a therapeutically actionable target in human disease. Biomolecules. 16:732. doi: 10.3390/biom16050732 PMC13204162 42194080

[ref65] Schiffmann S. N. Fisone G. Moresco R. Cunha R. A. Ferré S. (2007). Adenosine A2A receptors and basal ganglia physiology. Prog. Neurobiol. 83, 277–292. doi: 10.1016/j.pneurobio.2007.05.001, 17646043 PMC2148496

[ref66] Schiffmann S. N. Libert F. Vassart G. Vanderhaeghen J. J. (1991). Distribution of adenosine A2 receptor mRNA in the human brain. Neurosci. Lett. 130, 177–181. doi: 10.1016/0304-3940(91)90391-6, 1795877

[ref67] Sebastião A. M. Ribeiro J. A. (2023). Adjusting the brakes to adjust neuronal activity: Adenosinergic modulation of GABAergic transmission. Neuropharmacology 236:109600. doi: 10.1016/j.neuropharm.2023.109600, 37225084

[ref68] Shen H.-Y. Canas P. M. Garcia-Sanz P. Lan J.-Q. Boison D. Moratalla R. . (2013). Adenosine A2A receptors in striatal glutamatergic terminals and GABAergic neurons oppositely modulate psychostimulant action and DARPP-32 phosphorylation. PLoS One 8:e80902. doi: 10.1371/journal.pone.0080902, 24312250 PMC3842921

[ref69] Stöberl N. Maguire E. Salis E. Shaw B. Hall-Roberts H. (2023). Human iPSC-derived glia models for the study of neuroinflammation. J. Neuroinflammation 20:231. doi: 10.1186/s12974-023-02919-2, 37817184 PMC10566197

[ref70] Stockwell J. Jakova E. Cayabyab F. (2017). Adenosine A1 and A2A receptors in the brain: current research and their role in neurodegeneration. Molecules 22:676. doi: 10.3390/molecules22040676, 28441750 PMC6154612

[ref71] Terunuma M. Xu J. Vithlani M. Sieghart W. Kittler J. Pangalos M. . (2008). Deficits in phosphorylation of GABA A receptors by intimately associated protein kinase C activity underlie compromised synaptic inhibition during status epilepticus. J. Neurosci. 28, 376–384. doi: 10.1523/JNEUROSCI.4346-07.2008, 18184780 PMC2917223

[ref72] Thakur S. Du J. Hourani S. Ledent C. Li J.-M. (2010). Inactivation of adenosine A2A receptor attenuates basal and angiotensin II-induced ROS production by Nox2 in endothelial cells. J. Biol. Chem. 285, 40104–40113. doi: 10.1074/jbc.M110.184606, 20940302 PMC3000993

[ref73] Vien T. N. Ackley M. A. Doherty J. J. Moss S. J. Davies P. A. (2022). Preventing phosphorylation of the GABAAR β3 subunit compromises the Behavioral effects of neuroactive steroids. Front. Mol. Neurosci. 15:817996. doi: 10.3389/fnmol.2022.817996, 35431797 PMC9009507

[ref74] Wu G. Yu J. Wang L. Ren S. Zhang Y. (2018). PKC/CREB pathway mediates the expressions of GABAA receptor subunits in cultured hippocampal neurons after low-Mg2+ solution treatment. Epilepsy Res. 140, 155–161. doi: 10.1016/j.eplepsyres.2017.11.004, 29414524

[ref75] Xiao X. Wang Z.-Z. Xu W.-G. Xiao K. Cai Z.-Y. Sun X.-J. . (2012). Cerebral blood flow increase during prolonged hyperbaric oxygen exposure may not be necessary for subsequent convulsion. CNS Neurosci. Ther. 18, 947–949. doi: 10.1111/cns.12008, 23106977 PMC6493601

[ref76] Xie C.-w. Zhang Y.-n. Li R.-p. (2017). Interaction between adenosine and nitric oxide in the central nervous system and relationship with the therapy of epilepsy. Prog. Mod. Biomed. 17, 2996–3000.

[ref77] Yeung E. S. H. Tran D. T. Batchu S. N. Advani S. L. Liu Y. Kaur H. . (2025). Biphasic actions of the adenosine A2a receptor antagonist istradefylline in mice with obstructive uropathy. Sci. Rep. 15, 41110–41114. doi: 10.1038/s41598-025-24912-w, 41266603 PMC12635236

[ref78] You B. Shi G. Zhang Y. Fu X. Li Q. Wang Y. . (2025). The interaction of adenosine and dopamine in modulating the consequences of central nervous system oxygen toxicity. J. Appl. Physiol. 138, 169–181. doi: 10.1152/japplphysiol.00500.2024, 39601779

[ref79] Yu Z. S. N B. I. F P. T. S A. O. A G. N. T D. I. (2024). Mechanisms of monoamine oxidase involvement in the development of hyperbaric oxygen seizures. Zh. Evol. Biokhim. Fiziol. doi: 10.1134/S0022093024040161

